# Why Two? On the Role of (A-)Symmetry in Negative Supercoiling of DNA by Gyrase

**DOI:** 10.3390/ijms19051489

**Published:** 2018-05-16

**Authors:** Dagmar Klostermeier

**Affiliations:** Institute for Physical Chemistry, University of Muenster, Corrensstrasse 30, 48149 Muenster, Germany; dagmar.klostermeier@uni-muenster.de; Tel.: +49-251-83-23410

**Keywords:** type II topoisomerase, gyrase, DNA supercoiling, conformational dynamics, single-molecule Förster resonance energy transfer, domain communication

## Abstract

Gyrase is a type IIA topoisomerase that catalyzes negative supercoiling of DNA. The enzyme consists of two GyrA and two GyrB subunits. It is believed to introduce negative supercoils into DNA by converting a positive DNA node into a negative node through strand passage: First, it cleaves both DNA strands of a double-stranded DNA, termed the G-segment, and then it passes a second segment of the same DNA molecule, termed the T-segment, through the gap created. As a two-fold symmetric enzyme, gyrase contains two copies of all elements that are key for the supercoiling reaction: The GyrB subunits provide two active sites for ATP binding and hydrolysis. The GyrA subunits contain two C-terminal domains (CTDs) for DNA binding and wrapping to stabilize the positive DNA node, and two catalytic tyrosines for DNA cleavage. While the presence of two catalytic tyrosines has been ascribed to the necessity of cleaving both strands of the G-segment to enable strand passage, the role of the two ATP hydrolysis events and of the two CTDs has been less clear. This review summarizes recent results on the role of these duplicate elements for individual steps of the supercoiling reaction, and discusses the implications for the mechanism of DNA supercoiling.

## 1. Introduction

**Figure 1:**
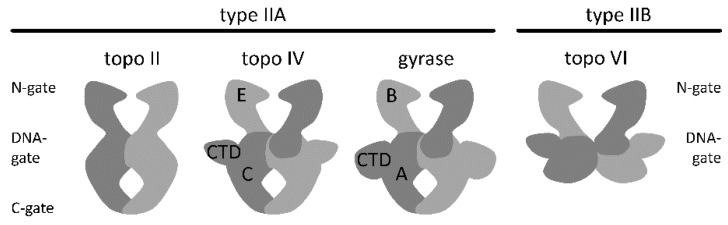
Architecture of type II topoisomerases. The type IIA topoisomerases topo II, topo IV and gyrase form a dimeric (topo II) or heterotetrameric structure with an N-gate, a DNA-gate and a C-gate. The type IIB topoisomerase topo VI is a heterotetramer that forms only two gates. A: GyrA subunit of gyrase, B: GyrB subunit of gyrase, C: ParC subunit of topo IV, CTD: C-terminal domains of GyrA (gyrase) and ParC (topo IV), E: ParE subunit.

DNA supercoiling affects DNA replication and recombination (reviewed in [1]), and gene expression [2,3]. The degree of supercoiling in vivo is regulated by DNA topoisomerases (reviewed in [4]). Members of the topoisomerase family catalyze the relaxation or introduction of negative or positive supercoils, or resolve catenanes (reviewed in [5]). These changes of the supercoiling state require DNA cleavage and re-ligation, mediated by conserved tyrosines [6]. Type I topoisomerases contain one catalytic tyrosine, and cleave one DNA strand. Type II DNA topoisomerases contain two catalytic tyrosines and can cleave both strands of the DNA substrate. Based on structural features, type II topoisomerases are subdivided into type IIA and type IIB enzymes. Type IIA enzymes form a symmetric structure with three protein/protein interfaces, termed N-gate, DNA-gate, and C-gate (Figure 1). Despite their common architecture, these enzymes catalyze different reactions in the cell: Eukaryotic topoisomerase II (topo II) catalyzes ATP-dependent DNA relaxation [7], bacterial gyrase mediates the ATP-dependent introduction of negative supercoils [8], and topoisomerase IV (topo IV) is responsible for ATP-dependent DNA decatenation [9]. All of these reactions are believed to occur via a strand-passage mechanism, guided by coordinated opening and closing of the three gates: First, a double-stranded DNA-segment, the G-segment (for gate), binds at the DNA-gate, and both DNA strands are cleaved. Closing of the N-gate, triggered by binding of ATP, then traps a second double-stranded DNA, the T-segment (for transport). DNA-gate opening enables the passage of the T-segment through the gap in the G-segment, leading to its relocation from the upper to the bottom cavity of the enzyme. The G-segment is re-ligated, and the T-segment leaves the enzyme through the C-gate. Topoisomerase VI, a type IIB topoisomerase, lacks the C-gate (Figure 1) and catalyzes ATP-dependent DNA relaxation by a hitherto uncharacterized mechanism involving only two gates [10,11].

The conformational changes of type II topoisomerases and their temporal coordination during catalysis are key for the mechanism of the reactions catalyzed, yet they are not well understood. In agreement with the strand-passage mechanism, cross-linking of the protein interfaces at the DNA- or C-gate of type II topoisomerases abrogates their activities [12,13,14]. However, the strand-passage event has never been observed experimentally.

Gyrase is the only type IIA topoisomerase that introduces negative supercoils into DNA in an ATP-dependent reaction. In bacteria, gyrase removes positive supercoils that accumulate ahead of replication forks [15] and ahead of RNA polymerase during transcription of active genes [16]. Gyrase is absent in humans, and thus serves as a drug target for therapeutics against bacterial and parasitic infections [17]. Severe side-effects and increasing resistance have led to a strong demand for improved gyrase inhibitors. Mechanistic studies on type IIA topoisomerases provide an important basis for the identification of novel and specific mechanism-based inhibitors for gyrase.

## 2. Architecture of Gyrase

**Figure 2:**
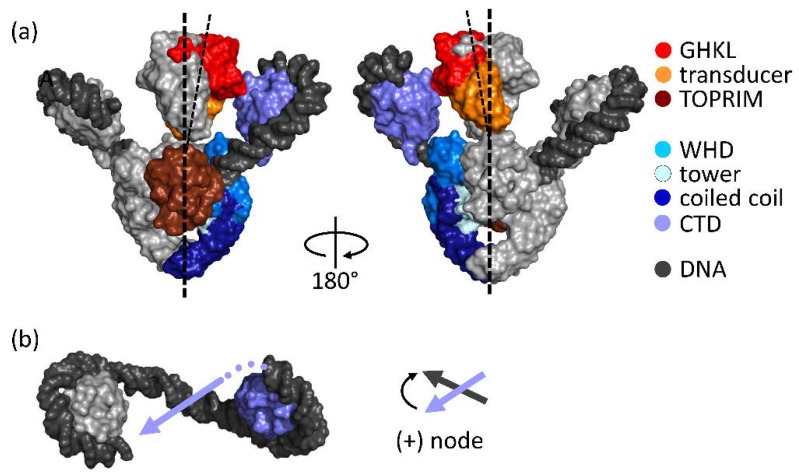
Domain organization and structure of gyrase bound to DNA. (**a**) Cryo-EM structure of *Thermus thermophilus* gyrase in complex with ADPNP, DNA, and ciprofloxacin [20] (surface representation, left: front view, right: back view). One GyrA and one GyrB subunit are depicted in a color code representing the individual domains, the second GyrA and GyrB subunits are shown in gray. GyrA consists of an N-terminal domain (NTD) and a C-terminal domain (CTD, violet). The NTD comprises the winged-helix domain (WHD, light blue) with the catalytic tyrosine, the tower domain (cyan), and the coiled coil domain (dark blue). GyrB consists of an ATPase domain of the GyrB-Hsp90-histidine/serine protein kinases-MutL (GHKL, red) family, a transducer domain (orange), and a topoisomerase-primase (TOPRIM) domain (dark red). The DNA is depicted in dark gray. The two CTDs of the GyrA subunits are in different positions with respect to the NTD dimer. The GyrB subunits, locked in the dimeric, nucleotide-bound state, are tilted away from the two-fold axis of the NTD dimer (broken lines), and are inclined towards the CTD that is positioned to guide the T-segment into the upper cavity between the GyrB arms (see also (**b**)); (**b**) Top view of the two CTDs and the DNA (dark gray) wrapped in a positive supercoil. The right-hand side CTD (violet) is positioned such that the exiting DNA (violet arrow) could serve as a T-segment. The T- and G-segment then form a positive (+) node. Dark gray arrow: G-segment.

Gyrase is a heterotetramer formed by two GyrA and two GyrB subunits (Figure 2). The GyrA subunits contain the catalytic tyrosines [6] that catalyze DNA cleavage and re-ligation [18,19]. The GyrB subunits provide the catalytic sites for ATP hydrolysis. The cryo-electron microscopy (cryo-EM) structure of *Thermus thermophilus* gyrase bound to the non-hydrolyzable ATP analog 5′-adenylyl-β,γ-imidotriphosphate (ADPNP), to DNA, and to the quinolone inhibitor ciprofloxazin has revealed the overall architecture of the enzyme [20] (Figure 2a). The gyrase N-gate is formed by the ATPase domains of GyrB. It is open in the absence of nucleotides [21,22] and closes the upper cavity upon ATP binding [21,22,23,24,25]. The DNA-gate at the center of the enzyme, jointly formed by GyrA and GyrB, separates the upper and lower cavities and contains the catalytic tyrosines for DNA cleavage. The C-gate at the bottom of gyrase delimits the lower cavity. The GyrA CTDs flank the sides of the gyrase body [26,27]. They form a 6-bladed β-propeller structure [28]. The CTDs bind DNA around their perimeter [29] and are important for wrapping of DNA around gyrase in a positive node (Figure 2b) [30,31].

## 3. Strand-Passage Mechanism for DNA Supercoiling

Gyrase is believed to catalyze DNA supercoiling by directional strand passage. Wrapping of the DNA around gyrase in a positive node by means of the CTDs (see Figure 2) [30,31] positions the G- and T-segments for strand passage [32]. T-segment capture upon ATP binding and N-gate closure, cleavage of the G-segment and opening of the DNA-gate then allow for strand passage and conversion of the positive DNA node into a negative node (sign inversion) [30,33,34], and a concomitant decrease in the linking number by two (+1 to −1).

**Figure 3:**
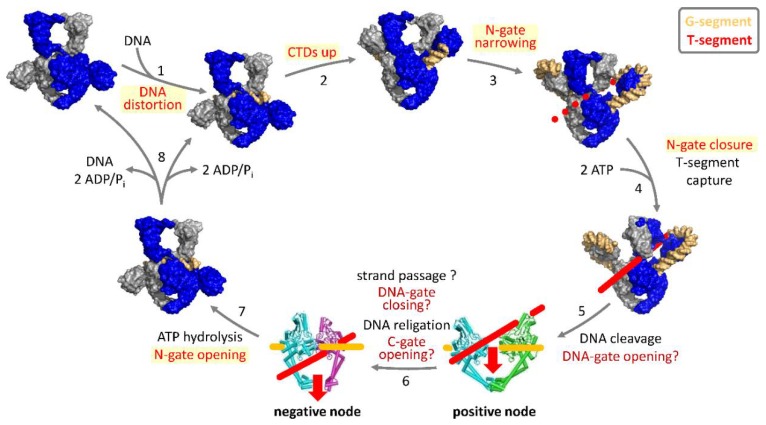
Strand-passage mechanism of negative supercoiling of DNA by gyrase and associated conformational changes. Gyrase binds the G-segment (orange) at the DNA-gate (1). DNA contacts with the CTDs, causing the CTDs to move upward (2). Wrapping of the DNA around the CTDs leads to narrowing of the N-gate (3). Nucleotide-induced N-gate closure fixes the T-segment (red) above the G-segment (4). Cleavage of the G-segment and DNA-gate opening (5) would then enable strand passage (6), which converts the DNA bound in a positive node into a negative node. DNA-gate closure allows the G-segment to be re-ligated. The T-segment could then exit from the lower cavity though the open C-gate (6). ATP hydrolysis leads to re-opening of the N-gate (7). After product release (8), gyrase is re-set for subsequent catalytic cycles. Postulated conformational changes are labeled in red, those demonstrated by single-molecule FRET are highlighted by a yellow box. DNA- and C-gate opening as well as strand passage have not been observed experimentally and are labeled with question marks. One GyrA and one GyrB subunit of the gyrase heterotetramer are depicted in blue, the second GyrA and GyrB subunits are shown in gray. The red dashed line in the gyrase/DNA complex after N-gate narrowing (3) indicates the extrapolated T-segment. In the crystal structures of GyrA dimers with open DNA- or C-gate (between steps 5 and 6, and 6 and 7, respectively), one GyrA subunit is shown in cyan, the second in green or magenta. The red arrows mark the passage of the T-segment through the gap in the G-segment and the open DNA-gate, and through the open C-gate, respectively.

Strand passage requires the coordinated opening and closing of the three gates of gyrase. Single-molecule Förster resonance energy transfer (FRET) is ideally suited to probe such large-scale conformational changes in the catalytic cycle of gyrase (reviewed in [35]). Single-molecule FRET experiments with donor/acceptor-labeled DNA substrates and donor/acceptor-labeled gyrase have revealed a cascade of DNA- and nucleotide-induced conformational changes during the supercoiling reaction. At the beginning of the catalytic cycle (Figure 3; reviewed in [36]), a G-segment binds at the DNA-gate of gyrase [37]. Binding is coupled to G-segment distortion and to DNA cleavage [37]. The CTDs contribute to distortion of the G-segment [37]. The DNA flanking the G-segment establishes contacts with the CTDs, which triggers their movement up and away from the GyrA NTD [27,38]. DNA wrapped completely around the CTDs then induces a narrowing of the N-gate [21]. DNA distortion and N-gate narrowing are linked to DNA cleavage [21,37]. In contrast, movement of the CTDs can occur independently of and possibly prior to cleavage [27]. ATP binding to the GyrB subunits causes N-gate closure [21,22], accompanied by a twisting motion of the GyrB subunits [20]. Closing of the N-gate fixes the T-segment above the G-segment in the upper cavity of gyrase. According to the strand-passage mechanism, the next steps are DNA-gate opening, strand passage, and C-gate opening to release the T-segment from the bottom cavity. Although structural data support that the DNA- and C-gates of gyrase can open in principle [39], we have not been able to detect DNA- or C-gate opening during the supercoiling reaction by single-molecule FRET. Hydrolysis of both ATP molecules leads to re-opening of the N-gate [21], and resets gyrase for subsequent catalytic cycles.

## 4. Symmetry, Asymmetry, and Inter-Subunit Communication in Gyrase

Due to its two-fold symmetry, the gyrase heterotetramer contains two active sites for ATP binding and hydrolysis, two CTDs for DNA wrapping, and two catalytic tyrosines for DNA cleavage. While the role for the two tyrosines appears evident from the need to cleave both strands of the G-segment for strand passage to occur, the role of the two CTDs is not immediately clear. The role of binding and hydrolyzing two ATP molecules for negative supercoiling of DNA has also been unclear until recently. Establishing a tandem-affinity purification procedure for heterodimeric gyrase [40] has paved the way to study asymmetric enzymes containing only one functional ATPase domain [41] one CTD [42], or one catalytic tyrosine [40]. The purified heterodimers are stable over extended periods of time because of the high stability of the GyrA dimer, so that subunit exchange and in situ formation of wildtype gyrase homodimers during supercoiling reactions does not occur [40,42].

### 4.1. Binding and Hydrolysis of a Single ATP is Sufficient for N-Gate Closure, Trapping of a T-Segment, and DNA Supercoiling

The role of the two ATP hydrolysis events for DNA relaxation by eukaryotic topoisomerase II has been dissected in detailed pre-steady-state kinetic studies of the wildtype enzyme [43,44], and by using affinity-purified heterodimers with one ATPase-deficient protomer [45]. These studies have revealed that topo II hydrolyzes ATP sequentially: A rapid hydrolysis step occurs before strand passage, and a slower hydrolysis event follows strand passage [43,44,45,46]. Heterodimeric topo II that can only hydrolyze one ATP per catalytic cycle is still able to couple ATP hydrolysis to DNA relaxation [45]. For *E. coli* gyrase, titration experiments with increasing fractions of a hydrolysis-deficient GyrB subunit and the resulting effect on DNA supercoiling have suggested that hydrolysis of a single ATP may support DNA supercoiling [47]. The function of the ATPase domains of GyrB can be manipulated in two different ways: Substitution of a glutamate that serves as the catalytic base and polarizes water to perform the nucleophilic substitution at the scissile bond by a glutamine renders the ATPase domain hydrolysis-deficient. This variant still binds ATP, although with reduced affinity [41,48,49]. Substitution of a conserved aspartate that interacts with the nucleobase by an asparagine, on the other hand, abolishes nucleotide binding [41,49,50].

**Figure 4:**
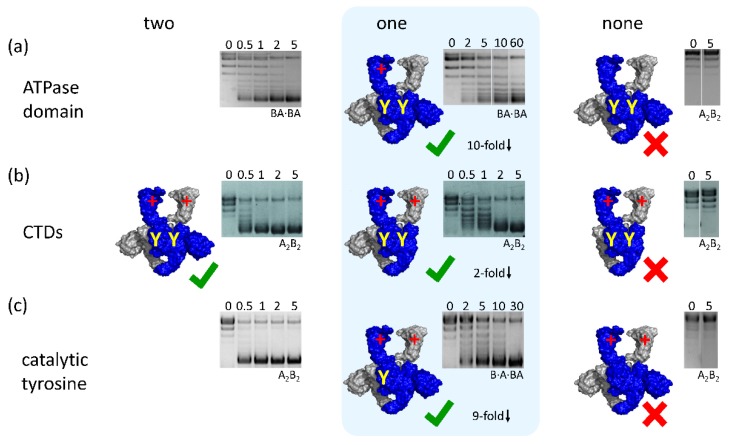
Negative supercoiling of DNA gyrase with a single catalytic tyrosine, a single functional ATPase domain, or a single CTD. (**a**) Gyrase that can only bind and hydrolyze a single ATP catalyzes negative supercoiling, although 10-fold more slowly than gyrase with two functional ATPase domains. Gyrase that cannot bind and hydrolyze ATP does not supercoil DNA. Data from Ref. [41]. Reprinted from *Journal of Molecular Biology* 429(23), Hartmann, S., Gubaev, A., Klostermeier D., Binding and Hydrolysis of a Single ATP Is Sufficient for N-Gate Closure and DNA Supercoiling by Gyrase, 3717-372., Copyright (2017), with permission from Elsevier; (**b**) Gyrase with a single CTD still catalyzes negative supercoiling of DNA. Supercoiling is two-fold slower than for wildtype gyrase. Gyrase lacking both CTDs does not supercoil DNA. Data from Ref. [42]; (**c**) Gyrase with a single tyrosine catalyzes negative supercoiling of DNA in the absence of strand passage. Supercoiling is about 9-fold slower compared with wildtype gyrase. Gyrase lacking both catalytic tyrosines is supercoiling-deficient. Data from Ref. [40]. Numbers above the lanes indicate the reaction times in minutes. A_2_B_2_: heterotetrameric gyrase, formed by two GyrA and two GyrB subunits; BA·BA: dimeric gyrase, formed by two GyrBA subunits; B·A·BA: heterotrimeric gyrase, formed by a GyrA·GyrBA heterodimer and GyrB. One GyrA and one GyrB subunit of the gyrase heterotetramer are shown in blue, the second GyrA and GyrB subunits are depicted in gray. The yellow Y marks the catalytic tyrosine. A red + indicates a functional ATPase domain in GyrB. The green tick marks highlight active enzymes, the red cross marks inactive species.

Gyrase that contains two hydrolysis-deficient GyrB subunits catalyzes a single round of DNA supercoiling, but is not capable of undergoing multiple turnovers [41,47,51]. Gyrase with two GyrB subunits deficient in ATP binding cannot supercoil DNA (Figure 4a). Gyrase that binds and hydrolyzes a single ATP can be generated by co-production of differentially tagged GyrB-GyrA fusion protein (GyrBA), one with an ATPase domain deficient in ATP binding and hydrolysis, and a second with a wildtype ATPase domain. Heterodimeric gyrase, carrying one of each tag, is then purified by tandem-affinity purification [40,41]. *Bacillus subtilis* gyrase that binds and hydrolyzes a single ATP can still supercoil DNA, although an order of magnitude more slowly and with reduced processivity [41] (Figure 4a). Its DNA-stimulated ATPase activity is about two-fold reduced compared to the wildtype enzyme with two catalytic sites [41]. Gyrase that binds and hydrolyses a single ATP closes the N-gate in response to nucleotide binding. The N-gate re-opens when the ATP is hydrolyzed, and the ADP·P_i_-bound state is generated [21,41]. Thus, the conformation of the N-gate is modulated with the nucleotide cycle of the single functional ATPase domain, whereas re-opening of the N-gate in wildtype gyrase requires hydrolysis of both ATP molecules bound. Nevertheless, the supercoiling efficiency of gyrase with a single functional ATPase domain is reduced compared with wildtype gyrase capable of binding and hydrolyzing two ATP molecules [41]. This difference in activities suggests that gyrase indeed catalyzes sequential hydrolysis of the two ATP molecules bound, as previously shown for topo II, and implies that an ATP/ADP·P_i_-bound state is populated during the catalytic cycle and is functionally relevant. If ATP hydrolysis by wildtype gyrase were concerted, the enzyme would switch from an ATP/ATP-bound state with a closed N-gate to an ADP·P_i_/ADP·P_i_-bound state with an open gate, much as the enzyme with only a single functional ATPase domain transits from the ATP-bound state with a closed N-gate to the ADP·P_i_-bound state with an open N-gate. Thus, similar supercoiling activities would be expected for both enzymes, which is contrary to the experimental results.

Why does binding and hydrolysis of a second ATP provide an advantage for supercoiling? It is conceivable that binding of the second ATP further stabilizes the GyrB dimer interface, and increases the efficiency of N-gate closing, T-segment capture, and DNA supercoiling [41]. It is also important to note that hydrolysis of the second ATP provides a timing function in the conformational cycle of DNA gyrase, which may be the key for coupling the nucleotide cycle to the supercoiling reaction [41].

### 4.2. Gyrase with a Single CTD Catalyzes Negative Supercoiling of DNA in Steps of Two

The role of the CTDs has been studied by characterizing the CTD individually, or by analyzing the effects of deleting both CTDs in gyrase on DNA binding, nucleotide- and DNA-driven conformational changes, DNA-stimulated ATPase activity, and DNA supercoiling [28,38,52,53,54,55]. Gyrase lacking the CTDs is not capable of introducing negative supercoils into DNA [21,56] (Figure 4). The variant cannot distort DNA bound at the DNA-gate [21], cannot wrap DNA in a positive node [56], does not show DNA-induced N-gate narrowing [21], and shows little DNA-stimulated ATPase activity [21,56].

What happens if only a single CTD is removed? Conceptually, DNA supercoiling by sign inversion according to the strand-passage mechanism should be possible with a single CTD: Binding of one CTD should be sufficient to stabilize the positive handedness of the crossing. Similarly, only one of the two CTDs can perform an active role in T-segment presentation in each catalytic cycle. Indeed, heterotetrameric *B. subtilis* gyrase containing GyrA heterodimers, formed by one subunit with, one without CTD, still catalyzes DNA supercoiling [42] (Figure 4b). The rate constant of supercoiling is only slightly reduced compared to wildtype enzyme [42]. DNA binding, distortion of the G-segment, and G-segment cleavage are unaltered by deletion of one of the two CTDs [42]. DNA binding induces an upward movement of the single CTD present. Gyrase with a single CTD also shows DNA-induced N-gate narrowing and DNA-stimulated ATP hydrolysis [42], demonstrating that inter-domain communication is intact. Most importantly, this enzyme also introduces negative supercoils in steps of two. Thus, a single CTD can stabilize DNA in a geometry suitable for supercoiling. Gyrase with a single CTD behaves very similar to gyrase with two CTDs, whereas gyrase lacking both CTDs is an entirely different enzyme that catalyzes different reactions.

### 4.3. Gyrase with a Single Catalytic Tyrosine: DNA Supercoiling in the Absence of Strand Passage

Double-strand cleavage by the two catalytic tyrosines is believed to be a central feature of the strand-passage mechanism of type II DNA topoisomerases, including gyrase. Cleavage-deficient gyrase in which both catalytic tyrosines are replaced by phenylalanines cannot catalyze DNA supercoiling [40,57] (Figure 4c). This variant does not distort DNA bound at the DNA-gate [37], does not respond to DNA binding by narrowing of the N-gate, and does not show DNA-stimulated ATPase activity [21]. On the other hand, cleavage-deficient gyrase is capable of undergoing DNA-induced displacement of the CTDs [27] and nucleotide-induced N-gate closure [21]. However, the N-gate also closes readily when nucleotide binds to the gyrase/DNA complex [27], whereas N-gate closure is hampered for cleavage-competent gyrase, presumably because a T-segment present between the two GyrB arms interferes with (ADPNP-induced) N-gate closing [21]. Thus, DNA cleavage seems to be a pre-requisite for T-segment capture.

Strikingly, *B. subtilis* gyrase with only a single catalytic tyrosine readily catalyzes negative supercoiling of DNA in an ATP-dependent reaction, despite the fact that it cannot catalyze double-strand cleavage and strand passage [40] (Figure 4c). The observed supercoiling activity can unambiguously be assigned to gyrase species with a single catalytic tyrosine, and is neither caused by subunit exchange and in situ generation of active species with two catalytic tyrosines nor by contaminations [40]. DNA supercoiling by gyrase containing only one catalytic tyrosine is also independent of construct design: Supercoiling was observed (1) with a heterotrimeric gyrase, obtained by co-production of a GyrBA fusion protein and a GyrA subunit carrying a Y→F mutation, rendering it cleavage-deficient, followed by tandem-affinity purification and re-constitution of gyrase by adding the second GyrB subunit; (2) with a heterodimeric gyrase, obtained by co-production of a GyrBA fusion protein and a GyrBA subunit carrying the Y→F mutation, followed by tandem-affinity purification; and (3) with heterotetrameric gyrase, obtained by co-production of GyrA and a GyrA subunit carrying the Y→F mutation, tandem-affinity purification and re-constitution of gyrase by adding GyrB [40]. *E. coli* gyrase with only a single catalytic tyrosine also catalyzes ATP-dependent negative supercoiling of DNA [40]. Most importantly, gyrase with a single catalytic tyrosine still changes the linking number of its DNA substrate in steps of two. 

**Figure 5:**
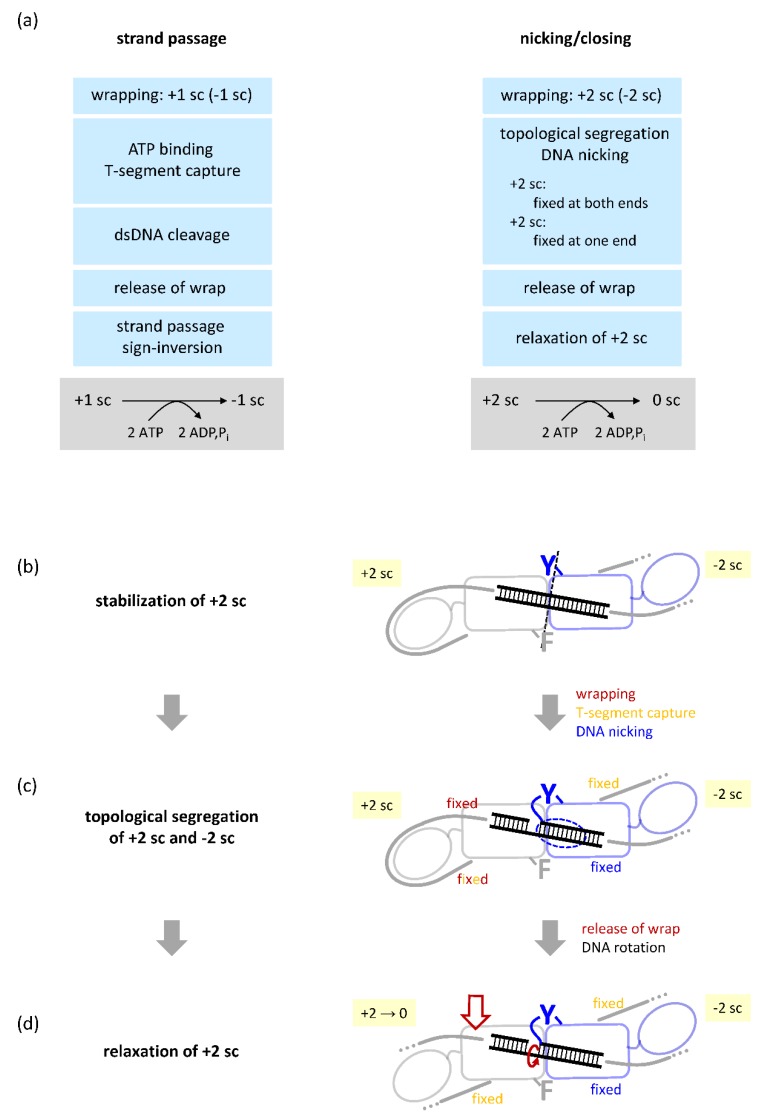
(**a**) Comparison of individual steps in negative DNA supercoiling by strand passage (left) and by capture, segregation, and selective relaxation of two positive supercoils in the absence of strand passage; (**b**–**d**) Model for DNA supercoiling by nicking-closing. The gray and blue rectangle represent the two WHD domains of the GyrA subunits in the top view; the ovals are the CTDs. The double-stranded G-segment is depicted in black; the DNA wrapped around the CTD is shown as a gray line. The catalytic tyrosine (blue Y) and the phenylalanine in a cleavage-deficient subunit (gray F) are highlighted. The GyrB subunits and the T-segment, situated above the G-segment, are omitted for clarity. (**b**) Binding of gyrase to DNA and DNA wrapping lead to the stabilization of two positive supercoils (+2 sc), associated with the formation of two negative supercoils (−2 sc) elsewhere in the DNA. (**c**) Topological segregation is achieved by fixation of the two sides of the G-segment, preventing rotation. Nicking (blue) prevents rotation of the DNA strand covalently attached to the catalytic tyrosine and of the base-paired complementary strand (dotted blue circle). The DNA exiting the right-hand side CTD (blue oval) is fixed because of fixation of the T-segment (orange). The DNA on the left-hand side of the nick is fixed by wrapping of the DNA around the perimeter of the second CTD (gray oval; red arrow), and by fixation of the adjacent T-segment by the closed N-gate (orange). (**d**) Release of the wrap on the left-hand side CTD (red open arrow) enables rotation of the G-segment around the non-cleaved strand (red circular arrow), which leads to spontaneous relaxation of the two positive supercoils (+2→0).

Gyrase containing a single catalytic tyrosine can only cleave one DNA strand [40], and DNA supercoiling by this enzyme thus cannot occur via the widely accepted strand-passage mechanism [33,34]. Therefore, we proposed an alternative mechanism for DNA supercoiling by this enzyme that is based on nicking and closing of DNA [40] (Figure 5). Nicking-closing mechanisms for gyrase action have been proposed previously [58,59,60], but were quickly rejected because they were not able to explain the decrease in linking number by two in each step of the catalytic cycle. 

Negative DNA supercoiling in steps of two would be possible through a sequence of trapping, segregating, and selectively relaxing two positive DNA supercoils [40] (Figure 5). When gyrase captures two positive supercoils in a covalently-closed circular DNA, two negative supercoils will form for compensation in the rest of the plasmid. Wrapping of the DNA around the CTDs fixes both ends of the G-segment emanating from the DNA-gate, preventing their rotation. N-gate closure upon nucleotide binding then also fixes the T-segment, leading to the segregation of positive and negative supercoils. On nicking of the DNA, one end of the G-segment remains fixed: The 5′-end of the nicked strand is covalently linked to the catalytic tyrosine, and the complementary strand is bound by base-pairing. On the other side of the nick, the DNA also cannot rotate because it is still wrapped around the CTD. This wrap is released upon ATP binding and N-gate closure [61]. On release of the DNA from the CTD, rotation around the bond in the non-cleaved strand becomes possible, allowing for spontaneous relaxation of the positive supercoils in this topological domain. After closing of the nick, the two negative supercoils remain, and the overall linking number has been reduced by two. This model for DNA supercoiling by nicking-closing predicts a critical role for only one of the two CTDs. In agreement with this prediction, and similar to gyrase containing two tyrosines, deletion of a single CTD does not abolish supercoiling in the context of gyrase with only one tyrosine [40]. The resulting enzyme still changes the linking number in steps of two [40]. The molecular details and possible structural basis of trapping two positive supercoils and their selective relaxation are currently unknown. 

Is DNA supercoiling by nicking-closing an alternative pathway or a path well-traveled? Supercoiling via nicking-closing might be a backup mechanism that is used only when double-strand cleavage and strand passage are not possible. However, it is conceivable that gyrase with two tyrosines can also follow this mechanism. Several lines of evidence support the hypothesis that nicking-closing might be a more general mechanism. Firstly, the succession of DNA- and ATP-induced conformational changes of gyrase at the beginning of the supercoiling cycle is identical, independent of the number of tyrosines present [40]. Secondly, DNA bound to gyrase is similarly distorted by gyrase containing one or two catalytic tyrosines [40], which suggests that this conformation reflects DNA with one strand cleaved. Thirdly, not only gyrase from *B. subtilis*, but also *E. coli* gyrase has the capacity to negatively supercoil DNA with a single catalytic tyrosine [40]. The capability of gyrases to supercoil DNA in the absence of strand passage would prevent double-strand breaks and genome instability.

Relaxation of two supercoils by rotation of the DNA in the gyrase/DNA complex requires substantial flexibility and plasticity of the protein–protein interfaces at the DNA- and C-gates. Cross-linking of these gates in *E. coli* gyrase, either with short cross-linkers or directly by oxidation of cysteines to a disulphide bond [13,14] abrogates DNA supercoiling activity. The loss of activity upon gate cross-linking is the strongest experimental evidence in favor of strand passage. However, such a tight cross-linking not only prevents gate opening but also severely restricts gate dynamics. Thus, it would presumably also prevent rotation of DNA that is required for supercoiling by nicking-closing [40]. The inhibition of DNA supercoiling by cross-linking is therefore not in contradiction with supercoiling by nicking-closing.

The observation of negative supercoiling activity with gyrase that carries only a single tyrosine raises the question whether other type II topoisomerase reactions can be catalyzed in the absence of strand passage. Gyrase with one tyrosine lost the ability to perform decatenation, a task that is performed in the cell by topo IV [9]. Gyrase lacking the CTDs is converted into a conventional topo II that catalyzes ATP-dependent DNA relaxation [56]. However, gyrase lacking the CTDs and containing only one catalytic tyrosine failed to catalyze ATP-dependent relaxation [40]. Thus, gyrase can catalyze its hallmark reaction, ATP-dependent DNA supercoiling, by nicking-closing, whereas decatenation (by topo IV) and ATP-dependent relaxation (by topo II) require double-strand breaks and strand passage. A comprehensive understanding of the mechanisms of type IIA topoisomerases therefore requires comparative mechanistic studies on gyrase, topo II, and topo IV, which are currently underway in our laboratory. 

### 4.4. A Minimal, Non-Redundant Gyrase? Implications for Inter-Domain Communication

If gyrase can catalyze DNA supercoiling by hydrolyzing a single ATP molecule, with one of the two CTDs, or with only one of its two catalytic tyrosines, is it possible to generate a minimal, non-redundant version of gyrase containing only one copy of each element? Gyrase with a single catalytic tyrosine that catalyzes DNA supercoiling by nicking-closing can also supercoil DNA coupled to hydrolysis of a single ATP [40]. Similarly, this enzyme can introduce negative supercoils into DNA in steps of two with just a single CTD [40]. These results suggest that supercoiling by a minimal gyrase with one functional ATPase domain, only one CTD, and a single catalytic tyrosine is possible. If this is the case, is there a particular spatial arrangement of these elements required? The preparation of heterodimeric gyrase offers the attractive possibility to probe the pathway of communication between individual domains of gyrase by introducing two modifications simultaneously. The introduction of the first modification renders the enzyme asymmetric. Any subsequent modification can now be introduced in two different configurations, either on the same side of the enzyme or on the opposite side. If a defined (asymmetric) pathway of communication exists, the effects of the second modification will be different, depending on the relative orientation with respect to the first. This is exemplified by gyrase carrying a single catalytic tyrosine that catalyzes DNA supercoiling by nicking-closing. According to the model for the mechanism of supercoiling by nicking-closing (Figure 5), the CTD on the cleavage-deficient subunit is required to capture two positive supercoils, whereas the CTD on the cleavage-competent GyrA subunit should be dispensable. In agreement with the model, deletion of the CTD on the cleavage-competent GyrA subunit does not abolish supercoiling [40]. These geometric considerations are also highly relevant for wildtype gyrase and its mechanism of supercoiling: The hydrolysis of the first ATP molecule, the capture of a T-segment with help of one of the CTDs, or cleavage of the first strand of the G-segment will naturally break the symmetry of the enzyme and render both sides of the enzyme mechanistically and functionally distinct. Asymmetry in gyrase/DNA complexes has been inferred from early footprinting studies of the bound DNA [31,62]. The cryo-EM structure of gyrase in the presence of ADPNP, ciprofloxazin, and DNA for the first time captured a structurally asymmetric state of gyrase and shows two distinct asymmetric features (see Figure 2): First, the two CTDs are in different positions, leading to different angles of the planes of the β-pinwheels with respect to the axis of the GyrA-NTD dimer. Only one of the two CTDs is suitably positioned to guide the T-segment into the upper cavity to stabilize a positive crossing of G- and T-segments. Second, the two inter-twined GyrB subunits bound to ADPNP are tilted away from the longitudinal axis of the enzyme, towards the CTD positioned for T-segment presentation. While it is currently unclear whether this structure reflects an on-pathway intermediate of the supercoiling reaction, it is tempting to speculate that these features may point to a role of asymmetry for gyrase function. The origin of the asymmetry in this structure, bound to ADPNP, is not immediately clear, though. It has been shown that dimerized GyrB can contain one or two molecules of ADPNP [63], and it is conceivable that the cryo-EM structure might represent an asymmetric state with only one ADPNP bound, and an empty nucleotide-binding site on the other GyrB subunit. The observed tilting of the dimerized GHKL domains of GyrB towards the potentially “active” CTD might then hint at nucleotide-dependent interactions between the ATPase domain and the CTD or the DNA bound to it. Clearly, further experiments are required to address the mechanism of communication between the ATPase domains and the CTDs. 

The order of events in the catalytic cycle, particularly the relation between cleavage of the two strands of the G-segment, presentation of a T-segment by one of the two CTDs, and hydrolysis of the two ATP molecules bound, is not entirely clear. The symmetry break might be coupled to cleavage of the first strand of the G-segment, leading to different propensities for DNA wrapping around the CTDs. This would then enable the interaction of one of the two CTDs with the now nearby ATPase domains, triggering the first ATP hydrolysis event. Alternatively, the trigger for breaking the symmetry could be the first ATP hydrolysis event, which then leads to an asymmetric conformation of the dimerized GyrB subunits and an asymmetric interaction with the now nearby CTDs that is selected as the “active” CTD. Finally, asymmetric interactions of the CTDs with the DNA flanking the G-segment on both sides might determine which strand is cleaved first, and which CTD interacts with the ATPase domains. Future studies therefore will not only have to address the pathway of communication between individual elements, but also the temporal sequence of individual steps in the catalytic cycle of gyrase.

## 5. Conclusions

DNA supercoiling by gyrase is supported by hydrolysis of a single ATP. Similarly, one CTD is required for DNA supercoiling, but the second is dispensable. Gyrase containing a single catalytic tyrosine can still supercoil DNA. All of these asymmetric enzymes catalyze DNA supercoiling in steps of two. While DNA supercoiling by gyrase with a single CTD or a single functional ATPase domain is compatible with supercoiling by sign inversion of a positive node of DNA through strand passage, supercoiling by gyrase with a single catalytic tyrosine must occur through nicking and closing of the DNA, and thus without strand passage. 

The supercoiling activity of gyrase containing a single functional ATPase domain, a single CTD, or a single catalytic tyrosine suggests that a minimal gyrase containing only one of each element, present in the correct geometry for productive inter-subunit communication, could catalyze DNA supercoiling. The pathway of communication between these elements is currently unknown, but can be dissected by inactivating two of these elements in combination in both relative configurations possible. 

The results summarized in this review have important implications for the identification of gyrase inhibitors: If supercoiling can occur with a single ATPase domain, a single CTD, or a single catalytic tyrosine, inhibitors need to target both active sites to achieve inhibition. Furthermore, it is currently unclear whether DNA supercoiling by nicking-closing is a back-up mechanism used by gyrase with a single catalytic tyrosine, or a more general pathway also used by wildtype gyrase. Mechanistic knowledge, particularly on conformational changes in the catalytic cycle of gyrase and on their temporal coordination, provides an important basis for the identification of mechanism-based conformationally selective gyrase inhibitors.

## Abbreviations

ADPNP	5′-adenylyl-β,γ-imidotriphosphate
CTD	C-terminal domain (of GyrA)
FRET	Förster resonance energy transfer
GHKL	GyrB-Hsp90-histidine/serine protein kinases-MutL
G-segment	gate segment
GyrA	GyrA subunit of gyrase
GyrB	GyrB subunit of gyrase
GyrBA	GyrB-GyrA fusion protein
NTD	N-terminal domain (of GyrA)
sc	supercoil
topo	topoisomerase
TOPRIM	topoisomerase-primase
T-segment	transport segment
WHD	winged-helix domain
